# Effect of Dimethylacetamide Concentration on Motility, Quality, Antioxidant Biomarkers, Anti-Freeze Gene Expression, and Fertilizing Ability of Frozen/Thawed Rooster Sperm

**DOI:** 10.3390/ani12202739

**Published:** 2022-10-12

**Authors:** Gamal M. K. Mehaisen, Ahmed M. Elomda, Shaimaa K. Hamad, Mona M. Ghaly, Yanyan Sun, Yunlei Li, Yunhe Zong, Jilan Chen, Agnieszka Partyka, Ali Nazmi, Ahmed O. Abbas, Farid K. R. Stino

**Affiliations:** 1 Department of Animal Production, Faculty of Agriculture, Cairo University, Giza 12613, Egypt; 2 Department of Animal Biotechnology, Animal Production Research Institute, Agriculture Research Center, Dokki, Giza 12572, Egypt; 3 Department of Animal Sciences, The Ohio State University, Columbus, OH 43013, USA; 4 Key Laboratory of Animal (Poultry) Genetics Breeding and Reproduction, Ministry of Agriculture and Rural Affairs, Institute of Animal Science, Chinese Academy of Agricultural Sciences, Beijing 100193, China; 5 Department of Reproduction and Clinic of Farm Animals, Faculty of Veterinary Medicine, Wroclaw University of Environmental and Life Sciences, 50-375 Wroclaw, Poland; 6 Food for Health Discovery Theme, The Ohio State University, Columbus, OH 43013, USA; 7 Department of Animal and Fish Production, College of Agricultural and Food Sciences, King Faisal University, Al-Ahsa 33843, Saudi Arabia

**Keywords:** chicken, semen freezing, dimethylacetamide, motility, fertility, antioxidant biomarkers, anti-freeze genes

## Abstract

**Simple Summary:**

Although sperm cryopreservation has a great potential for application in the poultry industry, it is still very limited at the economical scale due to the low fertility of frozen/thawed avian spermatozoa. Dimethylacetamide (DMA) has been widely employed as a cryoprotectant for the cryopreservation of chicken spermatozoa, but with variable results in motility and fertility. To determine the appropriate concentration of DMA in the chicken semen freezing extender, *in vitro* sperm quality and motility analyses and *in vivo* field trials based on the fertility of the frozen/thawed rooster sperm were conducted in the present study. The antioxidant biomarkers and novel anti-freeze-associated genes were also analyzed in the post-thawed sperm. Results conclude that DMA is preferably used at a low concentration to obtain applicable results in sperm motility and fertility after thawing without harming the quality and antioxidant system defense. The current study could highlight the possible molecular mechanisms behind the obtained fertility results of DMA-frozen sperm, especially when artificial insemination is implemented in the poultry industry.

**Abstract:**

Sperm cryopreservation is of great importance for the poultry industry but still needs to be optimized. The high susceptibility of poultry sperm to cryodamage leads to low fertility rates after cryopreservation. Therefore, the present study aimed at evaluating the effect of including a cryoprotectant, dimethylacetamide (DMA), in the chicken semen freezing extenders at a final concentration of 3%, 6%, or 9% on the post-thawed sperm motility, quality, antioxidant biomarkers, anti-freeze gene expression, and fertilizing ability. Results showed that the total motile sperm, progressivity, and viability were quadratically increased (*p* < 0.05) in the 6% DMA group. The antioxidant enzyme activity and lipid peroxidation were negatively (*p* < 0.05) affected by the increase in DMA concentration. Furthermore, some anti-freeze-associated genes such as heat shock protein 70 (*HSP70*) and ras homolog family member A (*RHOA*) were linearly and quadratically down-regulated (*p* < 0.05) with the high concentration of DMA. Finally, the fertility and hatchability rates did not indicate statistical differences between DMA groups. It can be concluded that using the low concentration of 3–6% DMA in the freezing semen extender is preferable to obtain acceptable results in the post-thawed sperm quality and fertility.

## 1. Introduction

The *ex-situ* “*in vitro*” conservation of animal genetic resources relies on the successful cryopreservation of germplasm components including gametes, embryos, and somatic cells [1]. In birds, sperm cryopreservation remains the most feasible technique for *ex-situ* management of avian genetic resources in addition to the great potential of application in the field of artificial insemination, disease prevention, breeding, and selection programs in the poultry industry [2]. The avian spermatozoa are known for their peculiar characteristics in morphology and functionality, such as the low quantity of cytoplasm, mitochondria, and antioxidants, and the high amounts of polyunsaturated fatty acids [3,4]. However, the spermatozoa maintain their viability and fertility in the female reproductive tract for several days [5]. These features increase susceptibility to damage and decrease fertility rates after cryopreservation of avian spermatozoa and, therefore, make the use of frozen/thawed semen very limited at an economical scale in the poultry industry [3]. 

*N,N*-Dimethyl acetamide (DMA), which consists of one amide group bound to two methyl groups [CH_3_C(O)N(CH_3_)_2_], is widely used as an intracellular cryoprotectant (CPA) during the freezing of avian sperm [2,6,7]. It has been reported that DMA has no d-orbital functions, has high cell membrane permeability, and is naturally involved in the biological process of cells; thus, it could yield applicable cryopreservation activity for sperm cells [8]. In addition, the contraceptive effects reported for other CPAs such as glycerol (GLY), and the need for removal before artificial insemination, does not exist with DMA [9]. It has been also reported that DMA toxicity could be less harmful when compared with the mechanical damage caused by GLY removal during the freezing of chicken semen, and thus DMA can be considered as a good alternative to GLY [10]. Other CPAs such as dimethyl sulfoxide (DMSO) and ethylene glycol (EG) have been also used for chicken semen cryopreservation. Compared with DMA, however, fertility results obtained from DMSO and EG are widely variant and depend on other factors such as concentration, diluent, and thawing temperature [11]. Although DMA has been considered as a popular cryoprotectant for chicken spermatozoa in many reports [7,12], it may have deleterious effects on frozen/thawed sperm [5]. For example, membrane fluidity and acrosome reaction of the cryopreserved sperm may be altered at the first contact with DMA and during post-thaw processing [13,14].

DMA freezing technology relies on its ability to penetrate the sperm and replace its intracellular water to prevent the damage caused by ice crystal formation during the freezing/thawing process [15]. In previous research, DMA has been included in semen extenders at a concentration ranging from 3% to 26%, either to examine or to improve the efficiency of chicken sperm cryopreservation with or without other supplementations [4,10,16,17,18,19,20,21,22]. The results of post-thawed sperm motility were appropriate around the level of 6% DMA concentration in different poultry species [21,22]. However, mean fertility obtained with cryopreserved chicken semen with DMA remains low and variable, ranging from 10% to 77% [10,20,23].

To the best of our knowledge, no studies have conclusively proved the optimal DMA concentration for chicken sperm cryopreservation in straws nor explained the possible mechanisms behind the obtained fertility results of DMA-frozen sperm. Therefore, the objective of the present study was to evaluate the effect of including DMA cryoprotectant in the chicken semen freezing extenders at different concentrations on post-thawed sperm motion, quality, and fertility parameters. Furthermore, the antioxidant biomarkers and the anti-freeze-associated gene expression were analyzed in the post-thawed sperm of DMA-frozen rooster semen.

## 2. Materials and Methods

### 2.1. Birds and Management

A total of twelve roosters (40-wk-old) of the Cairo-B2 chicken strain [24] and forty-five Arbor Acres hens (50-wk-old) were used for the present study. All chickens were placed in individual battery cages (50 × 50 × 60 cm) and maintained at the Agricultural Experiments Station (Cairo University) under the same environmental conditions (20–25 °C, 16L:8D photoperiod, and free access to water). Birds were fed a standard commercial breeder feed containing 14% crude protein, 2750 Kcal metabolizable energy, 4.15% crude fiber, and 3.3% crude fat (Feedmix-Egypt Co., Obour, Egypt).

### 2.2. Semen Collection and Freezing/Thawing Protocol

The roosters were subjected twice a week to an abdominal massage technique followed by a cloacal stroke to release the semen [25]. Individual semen ejaculates were received in sterilized glass tubes and immediately transferred into a water bath at 37 °C. An aliquot of 10 µL of the ejaculates was diluted with 490 µL of pre-warmed saline and sperm motility was evaluated, as mentioned later, using computer-assisted sperm analysis (CASA; SpermVision™ software, Minitube GmbH, Tiefenbach, Germany) with settings adjusted for rooster spermatozoa. Sperm concentration was estimated under a microscope at a magnification of 400× using a hemocytometer slide after dilution (1:200 *v*/*v*) with phosphate buffer saline (PBS) supplemented with 10% eosin solution (Bio-Diagnostic, Inc., Giza, Egypt). The ejaculates with at least 3 × 10^9^ sperm/mL and at least 60% progressive motility were selected and pooled for the present study. The pooled semen was then diluted at a ratio of 1:2 (*v*/*v*) with a basic EK extender developed by Łukaszewicz [26], and then divided into three equal groups. The diluted semen groups were cooled at 5 °C for 60 min. A pre-cooled dimethyl acetamide (DMA, Qualikems Fine Chem Pvt. Ltd., Vadodara, India) was then added gently to the diluted semen groups at a final concentration of 3%, 6%, or 9% and allowed equilibration at 5 °C for 10 min. After that, semen was loaded into 250 µL French straws (Minitube GmbH, Tiefenbach, Germany) and maintained over liquid nitrogen (LN_2_) vapor (approximately 5 cm above the LN_2_) for 15 min, then directly plunged into LN_2_. Straws were kept inside the LN_2_ for 3 months, then thawed in a water bath at 38 °C for 10 s.

### 2.3. Sperm Motion Characteristics 

An aliquot of 10 μL from the thawed samples in each treatment group was placed on a slide over a hot plate at 37 °C using the CASA system [27]. The motion characterization was recorded for each sample including the percentage of motile sperm cells (%), percentage of progressively motile spermatozoa (PROG, %), average path velocity (VAP, µm/s), curvilinear line velocity (VCL, µm/s), straight line velocity (VSL, µm/s), straightness (STR = VSL/VAP%), linearity (LIN = VSL/VCL%), wobble (WOB = VAP/VCL%), amplitude of lateral head displacement (ALH, µm), and beat cross frequency (BCF, Hz). A minimum of 5 microscopic fields (300–500 sperm per field) were randomly captured and analyzed using a negative high-contrast microscope (Olympus-BX, Tokyo, Japan) at 200× total magnification. The frame rate was set at 60 frames per second for the analysis. Sperm was classified as motile if VSL was >5 µm/s and classified as progressively motile if VAP was >20 µm/s and STR was >80%. 

### 2.4. Sperm Quality Characteristics 

#### 2.4.1. Viability

The percentage of viable sperm in each treatment group was evaluated using eosin-nigrosin stain as described in the previous study [11] with slight modification. Briefly, 10 μL thawed semen was mixed with 10 μL of 25% eosin-nigrosin staining solution (Bio-Diagnostic, Inc., Giza, Egypt), incubated at room temperature for 30 s, and smeared on a clean microscopic slide. The smear was allowed to air dry, and at least 200 sperm were examined under a phase-contrast microscope at a magnification of 1000× with oil immersion. Unstained spermatozoa were classified as live and pink-stained spermatozoa were classified as dead. 

#### 2.4.2. Acrosome Integrity

Sperm acrosome integrity was measured using Giemsa stain as described previously [28] with slight modification. In brief, 20 μL of thawed semen was smeared on a clean glass slide, dried, and then immersed in a fixative solution (100 mL formalin 35%, 9 g NaCl, and 12 g dibasic/anhydrous Na_2_HPO_4_ dissolved in 900 mL distilled water) for 15 min. The slide was then immersed for 90 min into a solution of Giemsa stain (Bio-Diagnostic) diluted with distilled water (1:4 *v*/*v*). The slide was washed under running tap water and allowed to dry at room temperature. At least 200 sperm were examined under 1000× magnification with oil immersion of a phase-contrast microscope. The intact sperm with blue-stained acrosomal caps were differentiated from unstained sperm in each sample for calculating the percentage of acrosome-intact sperm.

#### 2.4.3. Plasma Membrane Status

Sperm membrane integrity of frozen sperm after thawing was assessed through a hypo-osmotic swelling test (HOST) according to methods described by Rakha et al. [28] with slight modification. Briefly, 10 μL of thawed semen was mixed with 100 μL of pre-warmed swelling solution (0.75 g sodium citrate dihydrate and 1.375 g fructose in 100 mL distilled water, 100 mOsmol/kg) and incubated at 37 °C for 60 min. A drop (~20 μL) of the incubated solution was smeared on a clean glass slide then fixed in a formal saline solution for 15 min. At least 200 sperm were examined under a phase-contrast microscope (1000× with oil immersion). The spermatozoa showing swollen and coiled tails were classified as normal spermatozoa and were calculated as the percentage of spermatozoa having intact plasma membranes.

### 2.5. Sperm Antioxidant Biomarkers 

#### 2.5.1. Semen Extraction and Protein Assay

Six replicates per treatment group of about 0.4 mL each were obtained by thawing 2 straws of frozen semen per replicate. After thawing, each replicate sample was adjusted to 1 mL with PBS (pH = 7.4) in Eppendorf tubes (~400 × 10^6^ sperm/mL). Samples were washed twice with 1 mL PBS and precipitates were collected by centrifugation at 1030× *g* for 10 min at 4 °C. The final precipitates were resuspended in 1 mL of PBS containing 4% Triton X-100, incubated at room temperature for 30 min, and then centrifuged at 1030× *g* for 20 min at 4 °C. The supernatant was collected and stored at −20 °C for further analysis. 

The total protein was determined in each sample to normalize obtained data. The biuret reaction method [29] was performed according to the manufacturer protocol of the colorimetric assay kit (TP-2020, Bio-Diagnostic). In summary, 25 μL of the sample or the standard solution were incubated with 1 mL biuret reagent at 37 °C for 10 min. The absorbance of the sample (A_sample_) and standard (A_standard_) was read against the reagent blank at 550 nm using a scanning spectrophotometer (CE1010, Cecil Instruments Limited, Cambridge, UK). The protein concentration (g/dL) was calculated as (A_sample_/A_standard_ × 5).

#### 2.5.2. Total Antioxidant Capacity

A specific colorimetric kit (TAC-2513, Bio-Diagnostic) was used to determine the total antioxidant capacity (TAC) according to previously described methods [30]. Following the kit’s protocol, 20 μL of the sample was incubated with 500 μL of H₂O₂ substrate at 37 °C for 10 min. The mixture was then incubated with 500 μL of working chromogen reagent at 37 °C for 5 min. The absorbance of the blank reagent (A_blank_) and sample (A_sample_) against distilled water was read immediately at 505 nm. The TAC concentration was calculated as (A_blank_ − A_sample_ × 3.3) and expressed as μM/mg protein.

#### 2.5.3. Superoxide Dismutase Activity

The superoxide dismutase (SOD) activity was measured using colorimetric assay kits (SOD-2521, BioDiagnostic, Inc.). The assay relies on the ability of SOD enzyme to inhibit the reduction of nitro-blue tetrazolium (NBT) dye with NADH which is mediated by phenazine methosulfate (PMS) under aerobic conditions [31]. Briefly, 100 μL of the sample or control (distilled water) was mixed with 1 mL of working reagent (10 mL phosphate buffer pH 8.5, 1 mL NBT, and 1 mL NADH), then 100 μL of PMS was added to initiate the reaction. The increase in absorbance at 560 nm over 5 min was measured for the control (ΔA_control_) and for the sample (ΔA_sample_) at 25 °C. The SOD activity was calculated as unit/assay [U = (ΔA_control_ − ΔA_sample_)/ΔA_control_ × 100 × 3.75] and normalized per mg protein in each sample.

#### 2.5.4. Glutathione Peroxidase Activity

Glutathione peroxidase (GPx) activity was determined indirectly by measuring the consumption of reduced nicotinamide adenine dinucleotide phosphate (NADPH), according to the methods described in a previous work [32]. The assay was performed following the manufacturer protocol of the colorimetric kits (GPx-2524) obtained from BioDiagnostic, Inc. In brief, 10 μL of the sample was mixed with the reaction mixture containing 1 mL of assay buffer (phosphate buffer and Triton X-100, pH 7.0), 100 μL of reagent (glutathione, glutathione reductase and NADPH), and 100 μL of hydrogen peroxide (previously diluted 100 times). The decrease in absorbance at 340 nm per min (ΔA340) was recorded over a period of 3 min against deionized water. A convenient sample dilution was used to control the start of A340 at 1.5 and ΔA340 at 0.05 per min. The results of the GPx activity were expressed as units of GPx (ΔA340/0.00622 × dilution factor) and normalized per mg protein in each sample.

#### 2.5.5. Lipid Peroxidation

The level of lipid peroxidation (LPO) in the sample was indirectly measured by the analysis of malondialdehyde, using colorimetric assay kits (LPO-2529, Bio-Diagnostic), according to the methods described by Kei [33]. In brief, 200 μL of the sample or standard was heated in a boiling water bath with 1 mL of chromogen for 30 min. After cooling, the absorbance of the sample (A_sample_) against the blank and standard (A_standard_) against distilled water was measured at 534 nm. The LPO level in the sample was calculated as (A_sample_/A_standard_ × 10) and expressed as nM/mg protein.

### 2.6. Anti-Freeze-Associated Gene Expression

A quantitative real-time polymerase chain reaction (qRT-PCR) was performed in the present study for 4 selected anti-freeze associated genes [34], including heat shock protein 70 (*HSP70*), ras homolog family member A (*RHOA*), small nuclear ribonucleoprotein polypeptide A′ (*SNRPA1*), and ribosomal protein L29 (*RPL29*). Chicken glyceraldehyde 3-phosphate dehydrogenase (*GAPDH*) was chosen as a housekeeping gene to normalize the analytical variations of target genes. For the first step, primers of selected genes were designed using the Primer-BLAST web interface (http://www.ncbi.nlm.nih.gov/tools/primer-blast/index.cgi; accessed on 6 October 2022) and each pair of primers was tested to achieve efficiencies close to 1. The primers used are listed in Table 1.

Three replicates per treatment group (2 straws per replicate from different cryopreservation dates) and the control group (450 µL of fresh semen) were used for the qRT-PCR analysis. Samples were purified from somatic cells and immature spermatocytes as described previously with minor modification [35]. In brief, samples were completed to 1 mL with PBS then washed twice with PBS, as mentioned before. The final pellet was resuspended with 1 mL PBS and layered over 3 mL of pre-warmed (37 °C) density gradient medium (40% Pancoll human, density: 1.077 g/mL, PAN-Biotech GmbH, Aidenbach, Germany). Samples were then centrifuged at 300× *g* for 30 min at room temperature. The bottom layer was carefully transferred using a Pasteur pipette into a 2 mL cryovial and washed twice again with PBS. After that, the total RNA was extracted from the purified samples of fresh and frozen/thawed semen using the GeneJET™ RNA Purification Kit (Thermo Fisher Scientific, Waltham, MA, USA) following the manufacturer’s instructions. RNA was then transcribed into cDNA followed by PCR amplification using SuperScript^TM^ III One-Step RT-PCR System with Platinum^®^ Taq DNA Polymerase kit (Invitrogen, Thermo Fisher Scientific). To calculate the expression of selected genes, fold change was calculated using the 2^−ΔΔCt^ relative quantification method [36].

### 2.7. Fertility Trial

The Arbor Acres hens were divided into 3 equal groups (n = 15 per treatment group) for sperm fertility evaluation of the frozen/thawed semen with DMA. Each hen was inseminated 3 times at a 2 d interval with approximately 100 × 10^6^ sperm each time. Eggs were collected daily for each hen from the 2nd day of the first insemination until 2 days after the last insemination. Collected eggs were incubated for 21 days. At the end day of incubation, the number of hatched eggs was recorded. The unhatched eggs were opened and categorized into unfertile, early, or late mortality embryos, and pipped eggs.

### 2.8. Statistical Analysis

Six pools of semen ejaculates were selected per treatment group (n = 6) and considered as experimental units for all estimations of sperm motion characteristics, sperm quality characteristics, and antioxidant defense status, while three pools per treatment group (n = 3) were considered as experimental units for the estimation of anti-freeze gene expression. These variables were analyzed using one-way ANOVA with polynomial contrasts to explore the means, standard error of means (SEM), and the linear and quadratic trends of increasing DMA concentrations. A chi square test was performed to analyze the significance of fertility data between DMA groups. The differences were considered statistically significant at *p* < 0.05. All percentage data were normalized with an arcsine transformation. All data were analyzed using SPSS 22.0 (IBM corp., Armonk, NY, USA, 2013).

## 3. Results

### 3.1. Sperm Motion Characteristics 

Results of sperm motion characteristics as affected by freezing with DMA are shown in Table 2. The percentages of total motile sperm and PROG motility were quadratically increased (*p* < 0.05) in the 6% DMA group. No significant differences were found in sperm VAP, VCL, VSL, STR, LIN, WOB, and ALH among DMA concentration groups (*p* > 0.05). There was a linear decrease (*p* < 0.05) in the BCF as the DMA concentration increased. 

### 3.2. Sperm Quality Characteristics 

Results of sperm quality characteristics as affected by freezing with DMA are shown in Table 3. Sperm viability was quadratically increased (*p* < 0.05) in the 6% DMA group. It was found that DMA concentrations did not affect the integrity of sperm acrosome and plasma membrane.

### 3.3. Sperm Fertility Evaluation 

As shown in Table 4, DMA concentrations did not exert significant effects on fertility traits of post-thawed rooster sperm. It was observed that the percentage of hatched chicks was slightly lower, and the incidence of embryos’ early mortality in fertile eggs was slightly higher, although not significantly, in the 9% DMA group compared with the other DMA groups.

### 3.4. Sperm Antioxidant Biomarkers 

Results of antioxidant biomarkers in rooster sperm frozen with DMA are presented in Table 5. The DMA concentrations did not affect the TAC of frozen sperm. However, a linear and quadratic decrease (*p* < 0.05) was observed in the SOD and GPx activity when the DMA concentration increased. In contrast, a linear and quadratic increase (*p* < 0.05) was found in the LPO as the DMA concentration increased.

### 3.5. Anti-Freeze-Associated Gene Expression

**Figure 1:**
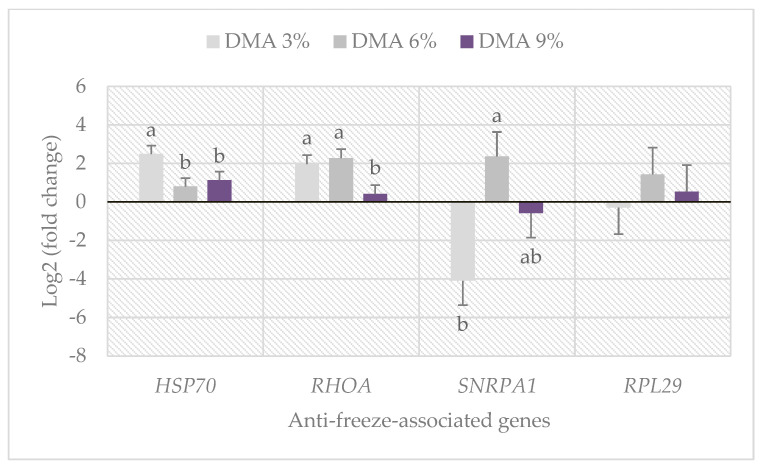
Effect of dimethyl acetamide (DMA) concentration in freezing extender on the anti-freeze-associated gene expression of rooster sperm after thawing. Bars express the means of three samples (n = 3) as log_2_ (fold change) ± standard error (SE). ^a–b^ Means with different superscripts, within the same gene, are significantly different (*p* < 0.05). *HSP70*: heat shock protein family A, *RHOA*: ras homolog family member A, *SNPA1*: small nuclear ribonucleoprotein polypeptide A′, *RPL29*: ribosomal protein L29. *p*-values of linear and quadratic effects are 0.021 and 0.038 for *HSP70*, 0.016 and 0.036 for *RHOA*, 0.032 and 0.005 for *SNPA1*, and 0.573 and 0.314 for *RPL29*, respectively.

The results of anti-freeze-associated gene expression of rooster sperm frozen with DMA are illustrated in Figure 1. The *HSP70* gene was linearly and quadratically down-regulated (*p* < 0.05) as the DMA concentration increased. The *RHOA* gene was linearly and quadratically up-regulated (*p* < 0.05) in the 3% and 6% DMA groups. The expression of *SNRPA1* gene was linearly and quadratically up-regulated (*p* < 0.05) in the 6% DMA group. There was no significant effect for DMA concentrations on the *RPL29* gene expression in post-thawed rooster sperm.

## 4. Discussion

DMA has been reported as one of permeating CPAs with accomplishment application in avian sperm cryopreservation [7,12]. However, it was reported that DMA concentration is a critical factor for recovering chicken sperm motility after thawing [22]. Results from the present study showed that the addition of DMA at a concentration of 6% to the freezing extender improved the post-thawed sperm motility and viability when compared with the other DMA concentrations (Table 2 and Table 3). In consistency with our results, Zaniboni et al. [22] indicated that motile sperm after pellet cryopreservation for chicken semen with 6% DMA was better than that obtained with 9% DMA, but they did not find a significant difference in the sperm viability due to DMA concentrations. In similar freezing protocols to that applied in the present study, the highest progressive motility and viability were recorded for the chicken semen frozen with 6% DMA compared with the lower concentrations of 2% and 4% DMA [37], or the higher concentration at 9% DMA [38]. Moreover, in the present study, no significant differences were observed between DMA concentrations in the integrities of sperm acrosome and plasma membrane. In contrast, other authors reported that higher DMA concentrations ranging from 9% to 12% induced a high progressive motility after thawing than lower DMA concentrations [39]. These results may support the suggestion that the CPAs, including DMA, have a concentration-dependent effect on the *in vitro* quality of frozen/thawed chicken sperm [37].

The DMA treatment did not exert statistically significant effects on the post-thaw sperm fertility traits. The decrease in fertilizing capacity of the frozen sperm with cryoprotectants, including DMA, may be attributed to more subtle damage than that occurring in the sperm motility, viability, acrosome structure, and plasma membrane integrity [37]. Moreover, it has been reported that sperm death and loss of motility cannot fully explain the reduction in the fertilizing ability of frozen sperm [40,41]. Our results indicated a linear and quadratic increase in the lipid peroxidation and decrease in the antioxidant enzyme activity in the sperm frozen with a high concentration of DMA (Table 5). The disturbance of antioxidant enzyme activity and high lipid peroxidation of the sperm may negatively affect the fertilizing ability of rooster spermatozoa [42]. This may also explain the high occurrence of early embryonic death and failed-hatching chicks with pipped eggs, although not significantly, in the 6% and 9% DMA treatment groups in the present study.

To the best of our knowledge, the current study is one of the first to explore the variation in chicken spermatozoal RNAs in response to cryodamage through analyzing the anti-freeze-associated gene expression in the DMA-frozen sperm. The thousands of predominantly nuclear-encoded transcripts have already been identified in the sperm of fertile chickens by using microarray analysis [35]. It was reported that transcripts in the sperm were varied according to the freeze/thaw cycle procedures, and subsequently this affected the fertilizing ability [43]. Qi et al. [34] recently explored a list of 2086 down-regulated genes and 29 up-regulated genes differentially expressed in frozen/thawed rooster sperm versus fresh sperm. Interestingly, the effect of freezing/thawing application on sperm’s fertilizing ability may be induced by modifying the expression of several genes associated with critical roles in cellular components, molecular functions, and biological processes [34]. Qi et al. [34], for example, found that *HSP70* and *HSP90* genes (famous members of the heat shock protein family) have been down-regulated after freezing/thawing treatment of rooster sperm. In addition, it was previously reported that HSPA8 (a highly conserved member of the *HSP70* family) could be used to improve the quality of cryopreserved sperm in bears [44] and in bulls [45]. In the present study, a significant decrease in the *HSP70* gene expression was obtained in the post-thawed rooster sperm frozen with 6 and 9% DMA groups compared with the 3% DMA group, while similar trends were observed in the antioxidant biomarkers for the same groups (Table 5). Thus, we speculate that *HSP70* has a cryoprotective effect like that reported for *HSP90* in sperm cryopreservation via eliminating the production of ROS [46] and/or reducing intracellular energy consumption [47].

*RHOA* is a small guanosine triphosphatase (GTPase) protein which has important roles in cellular biological processes, including cell motility, actin cytoskeleton regulation, and chromosome inheritance [48,49]. It was reported that *RHOA* expression by the qRT-PCR approach was positively correlated with the anti-freeze ability of cryo-dormant mouse embryos [50]. Moreover, it was found that *RHOA* gene expression was down-regulated after cryopreservation of rooster spermatozoa [34]. Our results are consistent with these reports indicating that *RHOA* was over-expressed in the 3% and 6% DMA groups where most of sperm characteristics were concomitantly good in the same groups in comparison with the 9% DMA group. Furthermore, *SNRPA1* is a spliceosome component, which belongs to the U2 small nuclear ribonucleoprotein A family and regulates the differentiation of spermatogonia into mature sperm [51]. We found that the *SNRPA1* gene, in contrast to the *HSP70* and *RHOA* genes, was markedly down-regulated in the 3% DMA concentration compared with the higher concentrations (6% and 9% DMA). The decrease in *SNRPA1* gene expression may be responsible for the decrease in sperm motility in the 3% DMA group since such correlation between hypermethylation of the *SNRPA1* gene and sperm motility has been previously reported [52]. Ribosomal proteins, including *RPL29*, might play an important role in sperm motility and fertilizing ability during cryopreservation [53,54]. In the study of Qi et al. [34], both *RPL31* and *RPL29* mRNA expression in rooster sperm were significantly decreased by the cryopreservation treatment, suggesting an effect of cold stress on sperm. However, the current study did not exert a significant effect for DMA concentrations on the *RPL29* gene expression in post-thawed sperm. Since other reports attributed the decrease in most gene transcripts to the freezing/thawing itself rather than the exposure to cryoprotectants [55], further molecular studies, such as RNA seq and protein expression, are required to verify such results for CPA effects on avian sperm cryopreservation.

## 5. Conclusions

Under the freezing protocol applied in the present study, sperm motility, progressivity, and viability were higher in the 6% DMA group compared with the 3 and 9% DMA groups. However, the fertility and hatchability rates did not indicate statistical differences between DMA groups, but were slightly higher in the 3% DMA group than in the 6% and 9% DMA groups. The post-thawed sperm antioxidant defense systems, including GPX, SOD, and LPO, were better in the 3% DMA group compared with the other groups. Moreover, some anti-freeze-associated genes such as *HSP70* and *RHOA* were highly expressed in the 3% DMA group, indicating a possible improvement in the sperm biological processes, compared with the higher DMA concentrations. Therefore, using DMA at a concentration of 3–6% in the freezing extender of chicken semen could be considered as an effective cryoprotectant to obtain acceptable results in the post-thawed sperm quality and fertility.

## Figures and Tables

**Table 1:** Primer sequences used for qRT-PCR analysis of selected anti-freeze-associated genes.

Gene	Gene Bank Accessing Number	Primer Sequences (5′-3′)	Product Size (bp)
*HSP70*	FJ217667.1	F-TTGATAAGGGCCAGATCCAG	105
		R-TGTTCAGCTCTTTGCCATTG	
*RHOA*	NM_204704.1	F-GAAGCAGGAGCCTGTCAAAC	132
		R-GCAGCTCTAGTGGCCATTTC	
*SNRPA1*	NM_001005823.1	F-CGACCTGCGGGGGTATAAAA	176
		R-GTCCTTCCCCAATCCGACAA	
*RPL29*	NM_001171677.1	F-GTCCCGTAAGTGGCACAGAA	157
		R-CTGCTTGGCATTGTTGGCTT	
*GAPDH*	NM_204305.1	F-AGAACATCATCCCAGCGTCCA	130
		R-CAGGTCAGGTCAACAACAGAG	

*HSP70*: heat shock protein 70; *RHOA*: ras homolog family member A; *SNRPA1*: small nuclear ribonucleoprotein polypeptide A; *RPL29*: ribosomal protein L29; *GAPDH*: glyceraldehyde-3-phosphate dehydrogenase.

**Table 2:** Effect of dimethyl acetamide (DMA) concentration in semen freezing extender on the motion characteristics of rooster sperm after thawing.

Parameters	DMA Concentration			SEM	*p*-Value		
	3%	6%	9%		Combined	Linear	Quadratic
Motile sperm (%)	59.1 ^ab^	64.9 ^a^	55.2 ^b^	2.62	0.008	0.161	0.004
PROG (%)	33.2 ^ab^	38.2 ^a^	30.5 ^b^	1.98	0.005	0.207	0.002
VAP (µm/s)	63.9	63.8	61.1	3.23	0.619	0.395	0.642
VCL (µm/s)	126.5	124.2	120.0	6.43	0.605	0.331	0.865
VSL (µm/s)	34.9	35.5	34.6	1.71	0.853	0.834	0.607
STR (%)	54.5	55.2	56.5	2.19	0.657	0.376	0.863
LIN (%)	27.8	28.3	28.8	1.64	0.832	0.551	1.000
WOB (%)	50.3	51.0	50.7	1.18	0.854	0.781	0.632
ALH (µm)	5.5	5.4	5.3	0.16	0.530	0.278	0.812
BCF (H_z_)	27.3	26.3	25.5	0.73	0.087	0.030	0.865

Data are presented as means ± standard error of means (SEM). Means with different superscripts, within parameter, are significantly different (*p* < 0.05). PROG: progressive motility; VAP: velocity average line (µm/s); VCL: velocity curved line (µm/s); VSL: velocity straight line (µm/s); STR: straightness (VSL/VAP%); LIN: linearity (VSL/VCL%); WOB: wobble (VAP/VCL%); ALH: amplitude of lateral head displacement (µm); BCF: beat cross frequency (H_z_).

**Table 3:** Effect of dimethyl acetamide (DMA) concentration in semen freezing extender on the quality characteristics of rooster sperm after thawing.

Parameters	DMA Concentration			SEM	*p*-Value		
	3%	6%	9%		Combined	Linear	Quadratic
Viability (%)	66.7 ^b^	85.6 ^a^	74.8 ^ab^	5.00	0.006	0.123	0.004
Intact acrosome (%)	85.9	89.4	87.4	2.61	0.438	0.574	0.253
Intact plasma membrane (%)	83.2	83.2	86.1	2.56	0.447	0.277	0.522

Data are presented as means ± standard error of means (SEM). Means with different superscripts, within parameter, are significantly different (*p* < 0.05).

**Table 4:** Effect of dimethyl acetamide (DMA) concentration in semen freezing extender on the fertility traits of rooster sperm after thawing.

Parameters	DMA Concentration			*p*-Value
	3%	6%	9%	
No. of incubated eggs	67	83	86	
Fertile eggs% (n) ^1^	28.4 (19)	18.1 (15)	23.3 (20)	0.327
Early embryo death% (n) ^2^	10.5 (2)	13.3 (2)	25.0 (5)	0.441
Late embryo death% (n) ^2^	5.3 (1)	0.0 (0)	5.0 (1)	0.670
Pipped eggs% (n) ^2^	42.1 (8)	53.3 (8)	45.0 (9)	0.800
Hatched eggs% (n) ^2^	42.1 (8)	33.3 (5)	25.0 (5)	0.527

^1^ Values were calculated as percentages of total incubated eggs. ^2^ Values were calculated as percentages of fertile eggs. (n): number of eggs per treatment group.

**Table 5:** Effect of dimethyl acetamide (DMA) concentration in semen freezing extender on the antioxidant biomarkers of rooster sperm after thawing.

Parameters	DMA Concentration			SEM	*p*-Value		
	3%	6%	9%		Combined	Linear	Quadratic
TAC (µM/mg) *	0.02	0.02	0.01	0.004	0.182	0.080	0.591
GPX (mU/mg) *	4.9 ^a^	1.1 ^b^	1.5 ^b^	0.383	<0.001	<0.001	<0.001
SOD (U/mg) *	22.0 ^a^	15.3 ^b^	13.6 ^b^	1.056	<0.001	<0.001	0.015
LPO (nM/mg) *	0.04 ^b^	0.23 ^a^	0.22 ^a^	0.046	0.001	0.001	0.025

Data are presented as means ± standard error (SE). Means with different superscripts, within parameter, are significantly different (*p* < 0.05). TAC: total antioxidant capacity, GPX: glutathione peroxidase, SOD: superoxide dismutase, LPO: lipid peroxidation. * Values were calculated per mg protein.

## Data Availability

Not applicable.
